# Evaluation of the Intervention Effect of *Bifidobacterium longum* on Constipation in Sleep-Deprived Mice

**DOI:** 10.3390/nu18182959

**Published:** 2026-09-09

**Authors:** Qimeng Liang, Xiaodong Song, Ran Wang, Yuanhong Xie, Hongxing Zhang, Junhua Jin

**Affiliations:** 1 Beijing Laboratory of Food Quality and Safety, Food Science and Engineering College, Beijing University of Agriculture, Beijing 102206, China; 2 Inner Mongolia Mengniu Dairy (Group) Co., Ltd., Key Laboratory of Dairy Quality Intelligence Monitoring Technology, State Administration for Market Regulation, Hohhot 011517, China; 3 Beijing Advanced Innovation Center for Food Nutrition and Human Health, China Agriculture University, Beijing 100083, China

**Keywords:** *Bifidobacterium longum*, sleep deprivation, constipation, gut–brain axis

## Abstract

Background/Objectives: It remains unclear whether sleep deprivation induces constipation. This study aimed to evaluate the intervention effects of *Bifidobacterium longum* 151-1 and BN-BB01 on constipation induced by sleep deprivation in mice, and to explore the underlying mechanism from the perspective of the gut-brain axis, so as to provide a theoretical basis for probiotic intervention in sleep-related gastrointestinal dysfunction. Methods: A mouse model of sleep deprivation was established via the multiple platform water environment method. Through the detection and analysis of relevant indicators, the effects of sleep deprivation on intestinal function in mice were systematically investigated, and the changes in various indicators of mice after *Bifidobacterium longum* intervention were observed. Results: Sleep deprivation significantly impaired intestinal motility and intestinal mucosal barrier function in mice, reducing fecal water content by approximately 21.4%, prolonging the time to first black stool defecation by about 57 min, and decreasing goblet cell count by nearly 50%. In terms of gastrointestinal hormones and neurotransmitters, sleep deprivation significantly downregulated the levels of excitatory gastrointestinal hormones (MTL, GAS) and excitatory neurotransmitter SP by 22.3%, 14.8%, and 16.2%, respectively. Additionally, sleep deprivation promoted the secretion of the inhibitory neurotransmitter VIP and stress hormone CORT, with their levels increased by 53.1% and 23.8%, respectively. The levels of neurotransmitters including 5HT and GABA were reduced by 15.6% and 23.2%, which ultimately led to a decrease in short-chain fatty acid (SCFA) levels. Intervention with *Bifidobacterium longum* significantly reversed the above abnormalities. It elevated SCFA levels and upregulated the expression of SCFA receptors GPR41 and GPR109A, promoted the expression of BDNF and GDNF, inhibited HDAC activity, increased the abundance of the beneficial bacterium *Akkermansia*, and reduced the abundance of the harmful intestinal bacterium *Alistipes*. Conclusions: *Bifidobacterium longum* can alleviate sleep deprivation-induced constipation in mice by regulating gut-brain axis function, optimizing intestinal flora structure, modulating the secretion of gastrointestinal hormones and neurotransmitters, and repairing the intestinal mucosal barrier. This study provides a solid theoretical basis for the clinical application of probiotics in the intervention of sleep-related gastrointestinal disorders.

## 1. Introduction

Constipation is a common digestive disorder characterized by difficulty in defecation, reduced stool frequency, and dry, hard stools [1]. Recent studies have found that psychosocial and emotional factors play a significant role in the development of constipation. A cross-sectional study on the risk factors for chronic constipation in adults showed that individuals with depression had a 1.97-fold higher risk of constipation than those without depression, indicating that depression significantly influences the occurrence of constipation [2]. There is a strong bidirectional association between negative emotions such as anxiety, depression, and chronic stress and constipation. Constipation that is significantly influenced by psychoneural regulation is termed “emotional constipation”. Emotional disorders can activate the hypothalamic–pituitary–adrenal (HPA) axis, affecting autonomic nervous and intestinal functions, and altering gastrointestinal motility. As a key neurotransmitter connecting the central and enteric nervous systems, 5-hydroxytryptamine (5-HT) not only regulates gastrointestinal motility but also plays a role in mood regulation, sleep, memory, and cognition [3], serving as an important link between emotion and constipation.

Sleep deprivation (SD) is one of the most critical factors affecting health [4], and can be considered a typical, persistent dual stressor—both emotional and physiological. Both long-term and short-term sleep deprivation can lead to gut microbiota imbalance, intestinal barrier damage, altered metabolite levels, and neurotransmitter disturbances [5,6]. Notably, there may be a potential link between sleep deprivation and constipation: insufficient sleep can inhibit intestinal peristalsis and disrupt intestinal epithelial integrity via HPA axis overactivation, vagal pathway abnormalities, and gut microbiota–brain axis signaling disturbances, thereby inducing constipation. However, most current constipation studies use drug-induced models, and research on sleep deprivation-induced constipation is limited. Therefore, this study adopts a sleep deprivation method to establish a constipation model for subsequent research.

Probiotics have garnered widespread attention due to their beneficial effects in regulating gut microbiota, protecting the intestinal barrier, reducing inflammation, and improving memory function [7]. It has been reported that the probiotic *Lacticaseibacillus rhamnosus* GG (LGG) can directly modulate gut microbiota structure to alleviate sleep deprivation-induced gut dysbiosis [8]. Therefore, improving sleep quality is crucial for preventing and alleviating intestinal damage and emotional disturbances.

Among various probiotics, the genus *Bifidobacterium* exerts beneficial functions by degrading dietary fiber to produce short-chain fatty acids (SCFAs), regulating immunity, and influencing neurotransmitter metabolism, thereby modulating the intestinal ecological environment and metabolic processes, and subsequently alleviating gastrointestinal discomfort. *Bifidobacterium longum*, a representative species of this genus, has shown marked effects in improving sleep disorders, relieving functional constipation, and regulating gut–brain axis function. Existing studies have shown that in patients with irritable bowel syndrome accompanied by depressive symptoms, certain probiotics exhibit mood-regulating potential; for example, *B. longum* NCC3001 significantly improved depression scores and reduced brain emotional reactivity [9]. *B. longum* can ameliorate stress-induced constipation in zebrafish under continuous light exposure by reducing cortisol levels and regulating intestinal inflammation [10]. However, no study has yet investigated the intervention effect of *B. longum* on a sleep deprivation-induced constipation model.

Based on this, the present study aims to establish a mouse model of emotional constipation induced by sleep deprivation, systematically investigate the intervention effects and underlying mechanisms of *B. longum* 151-1 and BN-BB01 from multiple dimensions and levels, and provide a theoretical basis for the future application of probiotics in sleep-related gastrointestinal dysfunction.

## 2. Materials and Methods

### 2.1. Experimental Strains

The bacterial strains used in the experiment were the identified preserved strains *B. longum* 151-1 (derived from mutagenesis of *Bifidobacterium longum* BB68S; GenBank: JBZDSS000000000) and BN-BB01 (CGMCC No.34432; also recorded in GenBank as strain 60-2, accession number JBZDSR000000000). Each strain was inoculated at a 2% inoculum size into nitrogen-flushed MRS liquid medium in anaerobic tubes, and cultured at 37 °C for 12 h. The same activation procedure was repeated as above.

### 2.2. Experimental Animals

A total of 60 specific-pathogen-free (SPF) 7-week-old male C57BL/6JNifdc healthy mice were used in this study, provided by Beijing Vital River Laboratory Animal Technology Co., Ltd. (Beijing, China). A total of 60 mice were used in this study and randomly allocated into five groups (*n* = 12 per group). The sample size was determined with reference to previously published studies using similar sleep-deprivation or constipation models in mice, and taking into account the statistical requirements for one-way ANOVA (α = 0.05, power ≈ 0.80). To allow for possible attrition, 12 animals were included in each group. In this study, mice were euthanized by cervical dislocation. The procedure was performed by personnel who had received professional training and passed competency assessment. Briefly, the base of the mouse’s skull was fixed with the thumb and forefinger of one hand, while the base of the tail was grasped with the other hand. A rapid backward pull on the tail was applied simultaneously with a forward push on the neck to quickly separate the cervical vertebrae from the skull, resulting in high cervical spinal cord transection and immediate death. Death was confirmed by palpation of clear separation of the cervical vertebrae, cessation of respiration and heartbeat, and absence of the corneal reflex. This method complies with the AVMA guidelines for the euthanasia of mice.

### 2.3. Experimental Methods

#### 2.3.1. Establishment of the Sleep Deprivation Model

Sixty SPF-grade, 7-week-old healthy male C57BL/6JNifdc mice were randomly divided into 5 groups (*n* = 12 per group) and acclimatized for one week with free access to food and water. The multi-platform water-environment apparatus consisted of a rectangular acrylic tank (50 cm × 30 cm × 25 cm) containing 20 platforms. The water level was maintained 1 cm below the platform surface. Mice could stand, move, and freely access food and water on the platforms; contact with water upon loss of muscle tone prevented them from entering rapid-eye-movement (REM) sleep. The control group was housed in standard cages. All groups of mice had free access to food and water.

Verification criteria for successful establishment of the sleep deprivation model: (i) During the daily 12-h intervention period, mice were observed for frequent falling into the water, immediate climbing back onto the platforms after falling, and standing while dozing. If a mouse remained inactive for more than 5 consecutive min or showed signs of sleep, it was awakened by gently tapping the tank wall to ensure that the animals remained awake throughout the entire sleep deprivation period. (ii) Defecation parameters of the mice were compared on day 14. The appearance of a constipation phenotype (significantly reduced fecal water content and prolonged time to the first black stool relative to mice housed in standard cages) confirmed successful model establishment.

**Figure 1:**
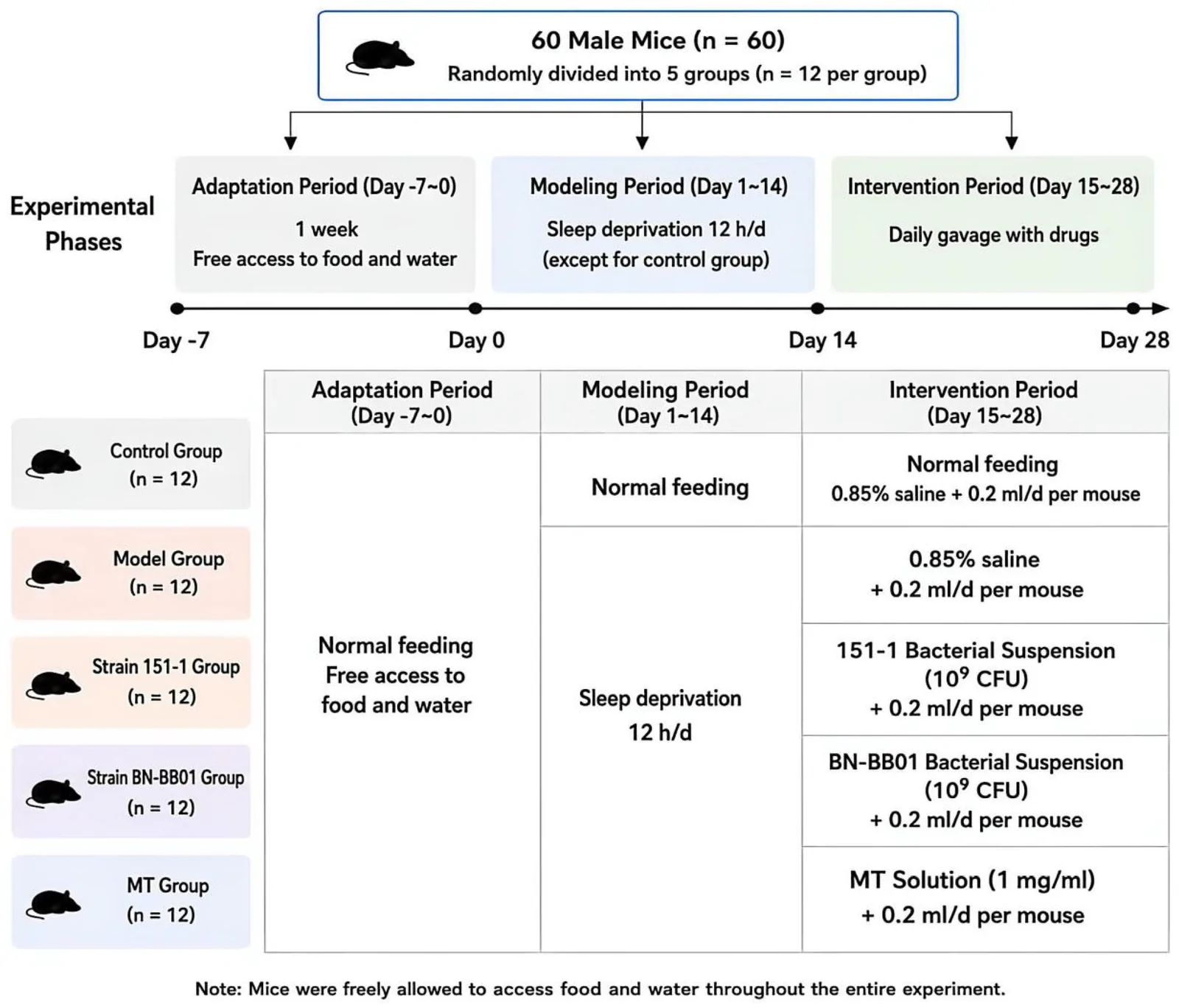
Flow diagram of animal experiments.

As shown in Figure 1, during the modeling period (days 1–14), mice in the control group were housed in standard cages, while the other four groups were placed in sleep deprivation cages for 12 h per day to establish a sleep deprivation-induced constipation model [11]. During this period, fecal water content and the time to first black stool defecation were measured to verify successful modeling. During the intervention period (days 15–28), the control group was housed normally and received a daily intragastric administration of 0.2 mL sterile saline. The model group was placed in a multi-platform water environment sleep deprivation apparatus for 12 h/d and received an equal volume of saline by gavage. The *B. longum* 151-1 and BN-BB01 groups, after sleep deprivation, were given 0.2 mL of the corresponding bacterial supernatant at a concentration of 10^9^ CFU/mL by gavage once daily. The positive control group, after the same sleep deprivation treatment, received 0.2 mL/d of melatonin solution at a concentration of 1 mg/mL by gavage.

#### 2.3.2. Determination of Apparent Constipation Indicators in Mice

On day 27, before gavage, fresh feces were collected directly into pre-weighed 1.5 mL centrifuge tubes within 3 min of defecation to minimize evaporative loss. The wet weight was immediately recorded using an analytical balance (NewClassic MF MS105DU, with a precision of 0.1 mg; Manufacturer: Mettler-Toledo AG, Greifensee, Switzerland). The tubes were then placed in an oven at 105 °C for 12 h. After cooling in a desiccator, the samples were weighed again [12], and this weight was recorded as the dry weight of the feces. Fecal water content (%) = (wet weight − dry weight)/wet weight × 100%.

On the morning of day 28, the mice were fasted for 12 h with free access to water. Each mouse was then administered 0.2 mL of activated charcoal suspension by gavage [12] and placed individually in a separate cage. The time was recorded. The timer was stopped when the mouse defecated its first black stool, and the interval was defined as the time to first black stool.

On the morning of day 29, the mice were gavaged with 0.2 mL of activated charcoal suspension containing *B. longum* 151-1, BN-BB01, or melatonin, respectively. After 30 min, the mice were sacrificed. The total length of the small intestine was measured, and the distance from the pylorus to the leading edge of the activated charcoal solution was recorded as the “charcoal propulsion distance” [13]. Small intestinal transit rate (%) = (charcoal propulsion distance/total small intestine length) × 100%.

#### 2.3.3. Histopathological Examination of Mouse Intestinal Tissue

Colon tissue samples were fixed in 4% paraformaldehyde solution for more than 24 h, then embedded in paraffin, sectioned, and stained with hematoxylin–eosin (HE) and periodic acid–Schiff (PAS).

#### 2.3.4. Determination of Gastrointestinal Hormones, Intestinal Motility, and Insomnia-Related Neurotransmitters in Mouse Serum

ELISA kits for gastrin (GAS, Cat. No. FY2857-A), motilin (MTL, Cat. No. FY2482-A), vasoactive intestinal peptide (VIP, Cat. No. FY2446-A), substance P (SP, Cat. No. FY2445-A), corticosterone (CORT, Cat. No. FY2061-A), serotonin (5-HT, Cat. No. FY2443-A), γ-aminobutyric acid (GABA, Cat. No. FY2442-A), and prostaglandin D_2_ (PGD_2_, Cat. No. FY43077-A) were purchased from Beijing Changhua Zhicheng Technology Co., Ltd. (Beijing, China). All assays were performed strictly in accordance with the manufacturer’s instructions, including standard dilution, sample loading, incubation, washing, enzyme addition, color development, reaction termination, and absorbance measurement.

#### 2.3.5. Determination of Short-Chain Fatty Acids in Mouse Feces

Fecal samples for SCFA analysis were collected, immediately snap-frozen in liquid nitrogen, and stored at −80 °C until analysis. For extraction, aliquots (0.1 g) of the frozen feces were weighed and thawed once at room temperature for 30 min (only a single freeze–thaw cycle was used, with no repeated cycles). After homogenization with a tissue homogenizer, the concentrations of short-chain fatty acids (including acetic acid, propionic acid, butyric acid, isobutyric acid, valeric acid, and isovaleric acid) were determined by gas chromatography (GC) according to the detection procedures and instrumental parameters described in reference [14].

The contents of SCFAs were determined by gas chromatography (GC) using the following conditions and temperature program. The GC system (7890A, Agilent Technologies, Palo Alto, CA, USA) was equipped with a flame ionization detector (FID) and a fused-silica capillary column (DB-WAX, 60 m × 0.25 mm i.d., 0.25 μm film thickness; Agilent). Helium was used as the carrier gas at a flow rate of 1 mL/min. The column oven temperature program was as follows: initial temperature 90 °C held for 1 min, then raised to 150 °C at a rate of 15 °C/min and held for 1 min, then further increased to 170 °C at 5 °C/min, and finally raised to 240 °C and held for 5 min. The injector and FID temperatures were set at 220 °C and 300 °C, respectively. The injection volume was 1 μL.

#### 2.3.6. Detection of Receptor Proteins in Mouse Colon Tissue

In this experiment, the expression levels of target proteins in mouse colon tissue samples were detected using Western blotting. First, total protein was extracted and the protein concentration was determined. SDS-PAGE gels of appropriate concentrations were prepared based on the molecular weights of the target proteins. After electrophoresis, the target proteins were thoroughly transferred onto PVDF membranes, followed by blocking and antibody incubation. Finally, the membranes were placed in a chemiluminescence imaging system to capture luminescent images. β-Tubulin was used as the internal reference protein, and the antibodies used were against GPR41 and GPR109A.

#### 2.3.7. Determination of Neurotrophic Factors and Epigenetic Regulators in Mouse Colon Tissue

The levels of histone deacetylase (HDAC, Cat. No. FY2679-B), brain-derived neurotrophic factor (BDNF, Cat. No. FY2204-B5), and glial cell line-derived neurotrophic factor (GDNF, Cat. No. FY2110-B) in mouse colon tissue were measured. The procedures were performed according to the kit instructions, including dilution of standards, sample addition, incubation, washing, enzyme addition, color development, termination, and measurement.

#### 2.3.8. Determination of Mouse Fecal Microbiota

The colonic contents of mice from the five groups were collected as samples for microbiome analysis. Total fecal DNA was extracted as a template following the instructions of the E.Z.N.A.^®^ soil DNA kit. The 16S V3-V4 rRNA gene was amplified using specific primers, and sequencing was performed on the Illumina NovaSeq platform. The DADA2 CCS sequence denoising method was used to process the optimized data to obtain amplicon sequence variant (ASV) representative sequences and abundance information. Based on the ASV representative sequences and abundance data, statistical analysis was performed on the Majorbio cloud platform.

#### 2.3.9. Statistical Analysis

In this study, GraphPad Prism 10.1.2 software was used. One-way analysis of variance (ANOVA) was applied to determine the statistical significance of differences between groups. *p* < 0.05 was considered statistically significant. ImageJ Version 1.54v software was used for quantitative analysis of the number of goblet cells in mouse colon histomorphology and the grayscale values of protein bands in Western blot analysis.

## 3. Results

### 3.1. Effect on Apparent Constipation Indicators in Mice

**Figure 2:**
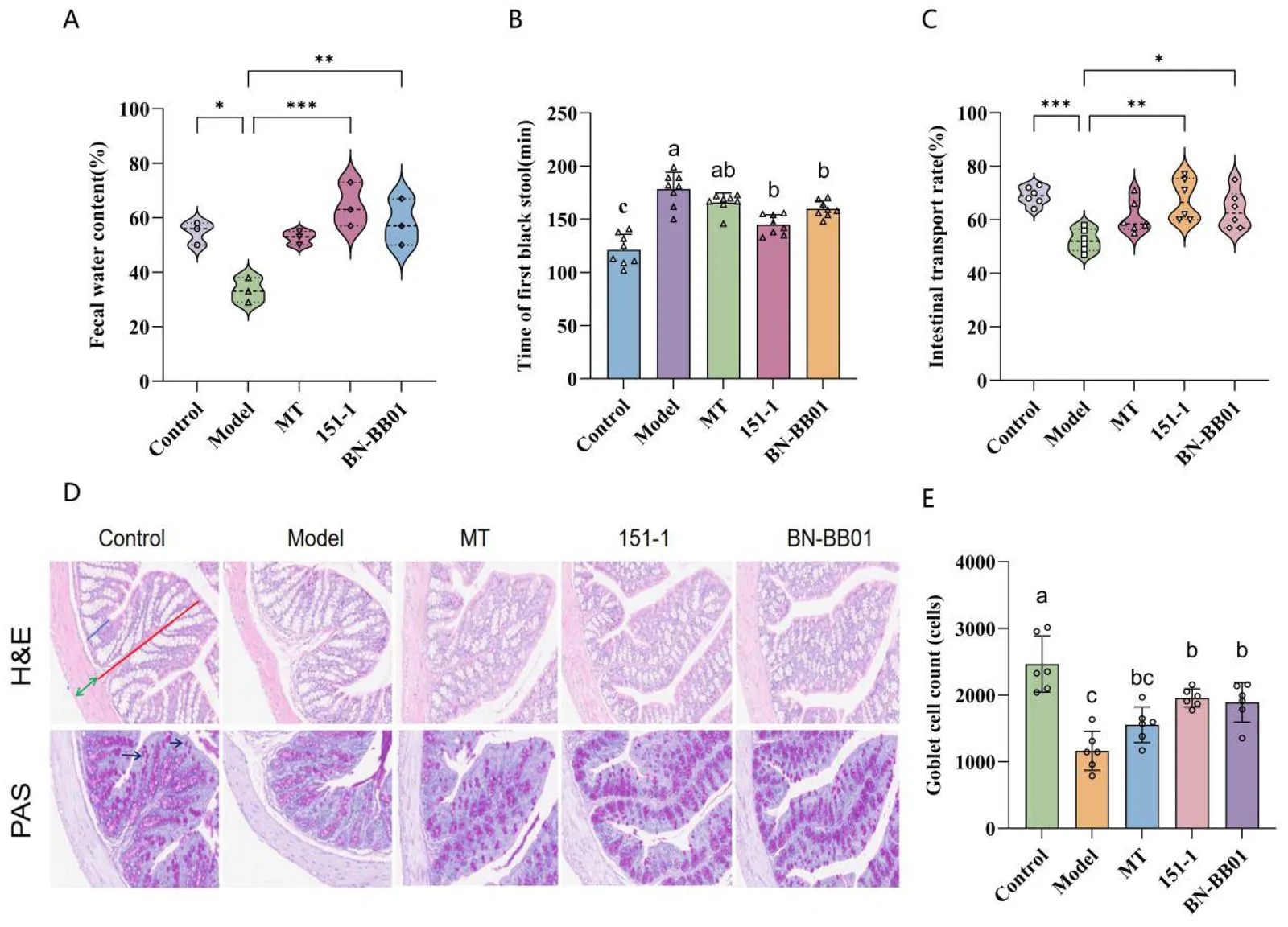
Apparent constipation indicators in mice: (**A**) Fecal water content, (**B**) time to first black stool defecation, (**C**) small intestinal propulsion rate, (**D**) histopathological analysis of colon tissue, (**E**) number of goblet cells in PAS staining. Note: In the figure, the red line indicates villus length; the blue line indicates crypt depth; the green line indicates muscle layer thickness; black arrows indicate goblet cells. Different lowercase letters under the same condition indicate statistically significant differences between groups (*p* < 0.05); * *p* < 0.05, ** *p* < 0.01, *** *p* < 0.001.

Fecal water content effectively reflects the softness or hardness of stools and is the most intuitive indicator for evaluating the relief of constipation. As shown in Figure 2A, the fecal water content of mice in the model group was significantly reduced by approximately 21.4% compared with the normal control group (*p* < 0.05), indicating that sleep deprivation induces a dry and hard constipation phenotype in mice. After intervention with *B. longum* 151-1 and BN-BB01, fecal water content was significantly increased compared with the model group (*p* < 0.001 and *p* < 0.01, respectively), whereas MT intervention showed no significant difference (*p* > 0.05). These results indicate that strains 151-1 and BN-BB01 effectively alleviate sleep deprivation-induced constipation, with an effect superior to that of the MT group.

The time to first black stool defecation directly reflects changes in gastrointestinal transit function. As shown in Figure 2B, the time to first black stool defecation in the model group was significantly prolonged by 57.00 min compared with the control group, an increase of 47.0% (*p* < 0.05), indicating that sleep deprivation successfully induced constipation symptoms and significantly inhibited intestinal motility in mice. After intervention with *B. longum* 151-1, BN-BB01, or MT, the time to first black stool defecation was significantly reduced compared with the model group (*p* < 0.05). These results indicate that strains 151-1 and BN-BB01 significantly ameliorate sleep deprivation-induced intestinal transit delay.

The small intestinal transit rate is a key phenotypic indicator for assessing intestinal motility and the degree of constipation, directly reflecting the transit efficiency of intestinal contents. As shown in Figure 2C, compared with the normal group, the small intestinal transit rate in the sleep deprivation model group was significantly reduced by 16.67 percentage points, a decrease of 24.2% (*p* < 0.001), suggesting that sleep deprivation leads to marked impairment of intestinal transit function. After intervention with MT, *B. longum* 151-1, or BN-BB01, the small intestinal transit rate was significantly restored. These results indicate that strains 151-1 and BN-BB01 effectively alleviate intestinal transit disorders in constipated mice, increase the small intestinal transit rate, reduce fecal transit time, and promote intestinal transport capacity.

### 3.2. Effects on Colonic Tissue Morphology in Mice

Sleep deprivation has a significant impact on the structural integrity of the intestinal barrier. HE staining results (Figure 2D) showed that in the healthy control group, the colonic mucosal tissue morphology was normal, villus length was appropriate, colonic smooth muscle layer thickness was within the normal range, and crypt structure was regular. In contrast, the model group exhibited thinning of the colonic smooth muscle layer, a marked reduction in villus length, and irregular crypt morphology, indicating that sleep deprivation-induced constipation leads to damage to the colonic mucosal barrier. In the *B. longum* 151-1 and BN-BB01 groups, villus morphology was improved, muscle layer thickness and crypt thickness were restored, and inflammatory cell infiltration in the lamina propria was reduced; the intervention effect was superior to that of the MT group. These results indicate that strains 151-1 and BN-BB01 effectively ameliorate sleep deprivation-induced intestinal barrier damage in mice.

Goblet cells, as the main cells responsible for intestinal mucus synthesis and secretion, play an important role in resisting external stimuli and maintaining the structural integrity of the intestinal barrier. PAS staining showed that in the control group, goblet cells were abundant and evenly distributed; the MT group had more goblet cells than the model group; the model group showed the most pronounced reduction in goblet cell number, with incomplete cell structures. After intervention with the 151-1 and BN-BB01 strains, these abnormalities were alleviated: the mucus layer on the mucosal surface was restored, goblet cell numbers increased, and this increase in goblet cells further improved the intestinal mucus barrier function, reduced resistance between the intestine and its contents, and thereby alleviated constipation symptoms.

Image J software was used to quantitatively analyze the number of goblet cells in PAS-stained sections. As shown in Figure 2E, the number of goblet cells in colonic tissue of the model group was significantly decreased compared with the normal control group (*p* < 0.05), with an average reduction of more than approximately 50%, indicating that the sleep deprivation-induced constipation model leads to disruption of the colonic mucosal mucus barrier and impaired peristalsis. After intervention with *B. longum* 151-1, BN-BB01, or the positive drug MT, the number of colonic goblet cells was significantly increased compared with the model group (*p* < 0.05). Among these, the 151-1 and BN-BB01 intervention groups showed better effects, with the average goblet cell count restored to approximately 2000, indicating a marked overall recovery.

### 3.3. Effects on Serum Levels of Gastrointestinal Hormones and Intestinal Motility Neurotransmitters in Mice

**Figure 3:**
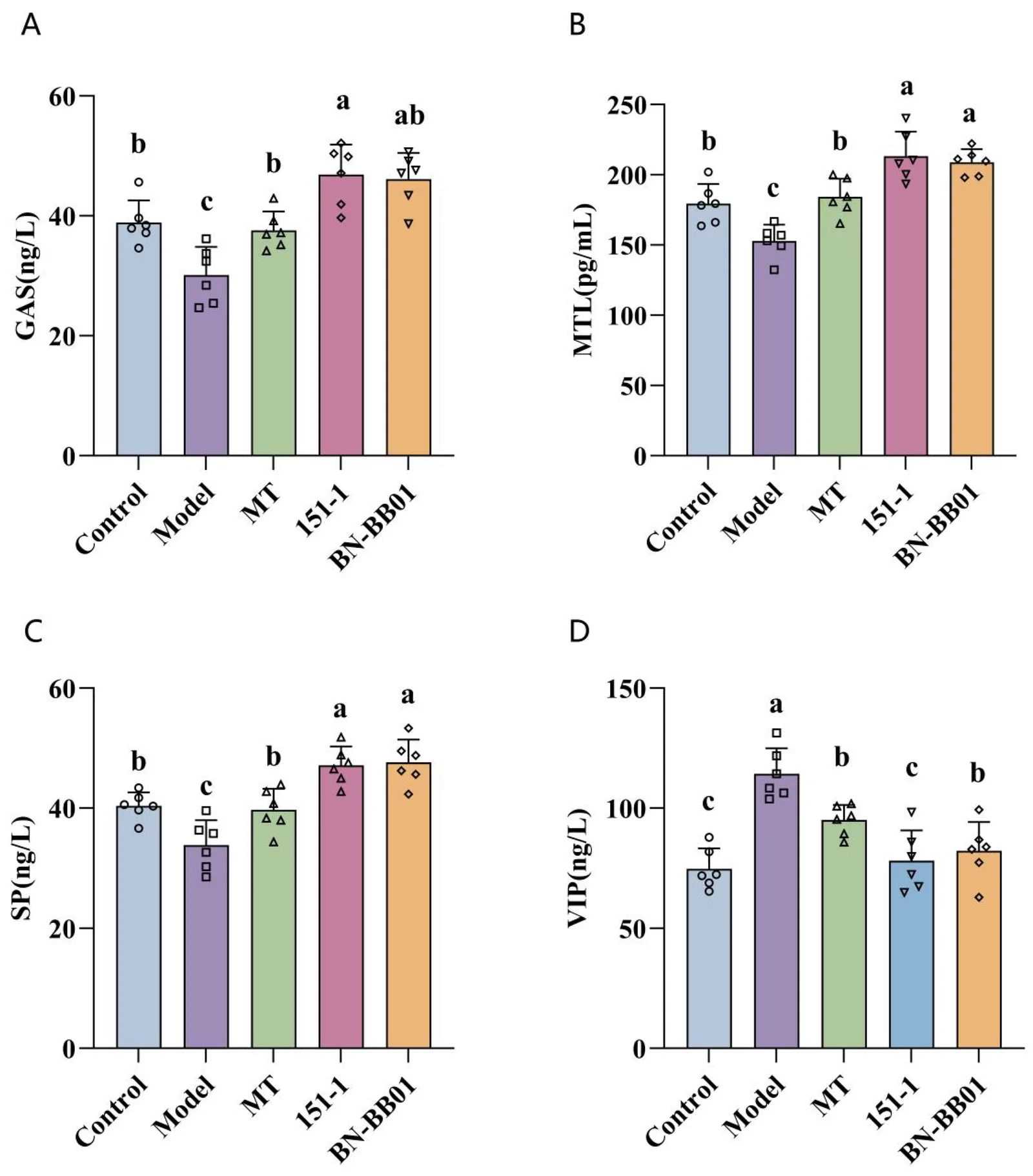
Levels of gastrointestinal hormones and intestinal motility indexes: (**A**) GAS content, (**B**) MTL content, (**C**) SP content, (**D**) VIP content. Different lowercase letters indicate significant differences among groups (*p* < 0.05).

Both motilin (MTL) and gastrin (GAS) are excitatory gastrointestinal hormones [15] that promote gastrointestinal smooth muscle contraction and play important regulatory roles in maintaining normal intestinal peristalsis. As shown in Figure 3A,B, compared with the Control group, serum GAS levels in the Model group decreased by approximately 8.67 ng/mL, a reduction of 22.3% (*p* < 0.05), and MTL levels decreased by 26.53 pg/mL, a reduction of approximately 14.8% (*p* < 0.05), indicating that sleep deprivation-induced disruption of gastrointestinal hormone secretion inhibits intestinal peristalsis. After intervention with *B. longum* 151-1 and BN-BB01, serum GAS and MTL levels were significantly increased (*p* < 0.05), and the effects were superior to those in the Control and MT groups. Taken together, these results indicate that *B. longum* 51-1 and BN-BB01 interventions effectively reverse the sleep deprivation-induced decrease in serum GAS and MTL levels, upregulate gastrointestinal hormone secretion, and promote intestinal peristalsis, whereas melatonin solution did not show a significant regulatory effect.

Intestinal motility disorders mediated by an imbalance in the enteric neural regulatory network are one of the mechanisms underlying constipation. The enteric nervous system (ENS) maintains normal peristalsis and smooth muscle contraction by regulating excitatory and inhibitory neurotransmitters [16]. The inhibitory neurotransmitter vasoactive intestinal peptide (VIP) and the excitatory neurotransmitter substance P (SP) were selected as indicators. As shown in Figure 3C,D, SP levels in the Model group were significantly lower than those in the Control group, with a reduction of approximately 16.2% (*p* < 0.05), indicating that the secretion of prokinetic neuropeptides was inhibited under sleep deprivation conditions. After intervention with strains 151-1 and BN-BB01, SP levels were significantly higher than those in the Model group, increasing by 13.27 ng/L and 13.80 ng/L, respectively (*p* < 0.05), and were also significantly higher than those in the Control group and the positive drug MT group (*p* < 0.05), indicating a positive correlation between increased SP secretion and *B. longum* intervention. Compared with the control group, VIP levels in the model group were significantly increased, with an elevation of approximately 53.1% (*p* < 0.05), indicating that overactivation of inhibitory neurotransmitters in the sleep deprivation-induced constipation model inhibits intestinal peristalsis. After intervention with *B. longum* 151-1 and BN-BB01, VIP expression levels were significantly reduced by 36.17 ng/L and 32.09 ng/L, respectively (*p* < 0.05), and the 151-1 group was closer to the control group level, indicating that *B. longum* can alleviate constipation by promoting small intestinal smooth muscle contraction through the regulation of gastrointestinal neuroendocrine function.

### 3.4. Effects on Serum Levels of Insomnia-Related Neurotransmitters in Mice

**Figure 4:**
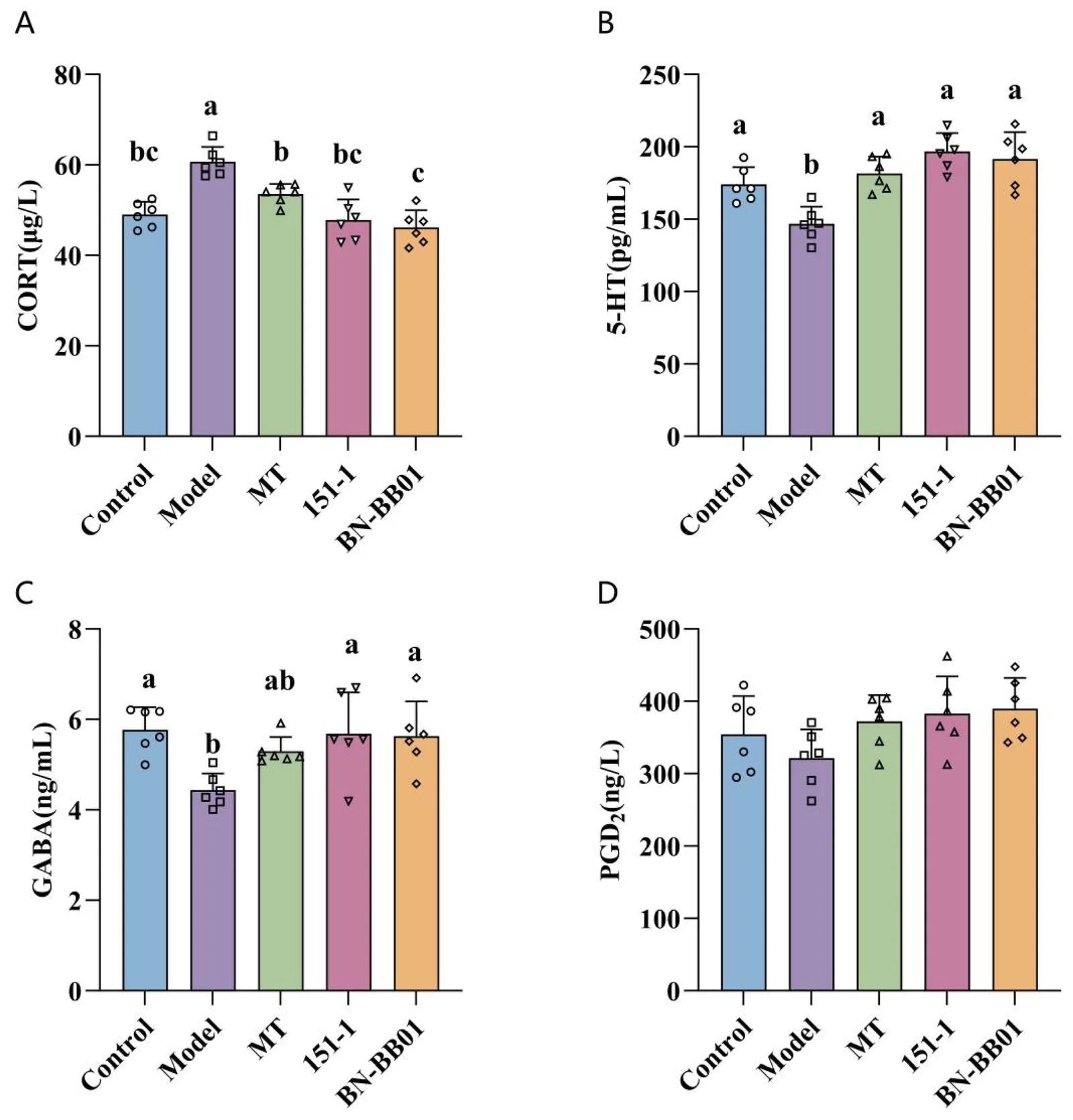
Levels of insomnia-related neurotransmitters: (**A**) CORT content, (**B**) 5-HT content, (**C**) GABA content, (**D**) PGD_2_ content. Different lowercase letters indicate significant differences among groups (*p* < 0.05).

Cortisol (CORT), as a stress hormone, can lead to secretory dysregulation under conditions of emotional stress, thereby inhibiting intestinal motility through the gut–brain axis [17]. Measuring this indicator can determine the role of stress hormones in constipation and the regulatory targets of probiotic intervention. As shown in Figure 4A, CORT levels in the Model group were significantly higher than those in the Control group, with an increase of approximately 11.68 μg/L, representing about a 23.8% elevation (*p* < 0.05), indicating that sleep deprivation leads to hypersecretion of CORT, which may be one of the causes of emotional constipation. Compared with the model group, the 151-1 group showed a reduction of approximately 12.88 μg/L (*p* < 0.05), and the BN-BB01 group showed a reduction of 14.55 μg/L (*p* < 0.05). The levels in both intervention groups were significantly lower than those in the model group and close to those in the control group, whereas the MT group did not show a significant regulatory effect. These results suggest that the mood-ameliorating effect of *B. longum* 151-1 and BN-BB01 may be achieved by downregulating CORT levels and modulating HPA axis activity.

To investigate the effect of *B. longum* 151-1 and BN-BB01 on alleviating anxiety in sleep-deprived mice, several common neuroactive substances were selected. Neurotransmitters such as 5-hydroxytryptamine (5-HT), γ-aminobutyric acid (GABA), and prostaglandin D_2_ (PGD_2_) are indispensable for sleep regulation. PGD_2_ plays a role in regulating sleep and central nervous system homeostasis [18]. 5-HT, an important neurotransmitter regulating sleep and mood, is synthesized predominantly (about 90%) in the gut, and the gut microbiota can influence 5-HT production by modulating tryptophan metabolism and enterochromaffin cell activity [19]. GABA, as a major inhibitory neurotransmitter in gut–brain axis regulation, can lead to an imbalance in enteric neural regulation when its secretion is disturbed, while also affecting the transmission of central stress signals to the gut [20]. As shown in Figure 3B and 3C, 5-HT and GABA levels in the Model group were significantly lower than those in the control group, decreasing by 27.12 ng/mL and 1.34 ng/L, respectively, with reductions of approximately 15.6% and 23.2% (*p* < 0.05), indicating that neurotransmitter metabolic levels are dysregulated in sleep-deprived mice. After intervention with strain 151-1, 5-HT and GABA levels were significantly increased, with elevations of 33.9% and 28.2%, respectively (*p* < 0.05); the BN-BB01 strain also increased 5-HT by 44.71 pg/mL and GABA by 1.20 ng/mL. Although PGD_2_ levels did not change significantly, they were still slightly higher (approximately 65 ng/L) than those in the model group (Figure 4D). The positive drug MT showed a slightly weaker effect than the probiotic strains in modulating the expression of these neurotransmitters, but it still alleviated the imbalance in insomnia-related neurotransmitters compared with the sleep-deprived model group. These results indicate that *B. longum* 151-1 and BN-BB01 can alleviate sleep deprivation-induced constipation in mice by regulating both excitatory and inhibitory sleep-related neurotransmitters in serum, thereby promoting intestinal peristalsis and smooth muscle relaxation.

### 3.5. Effects on Fecal Short-Chain Fatty Acids in Mice

**Figure 5:**
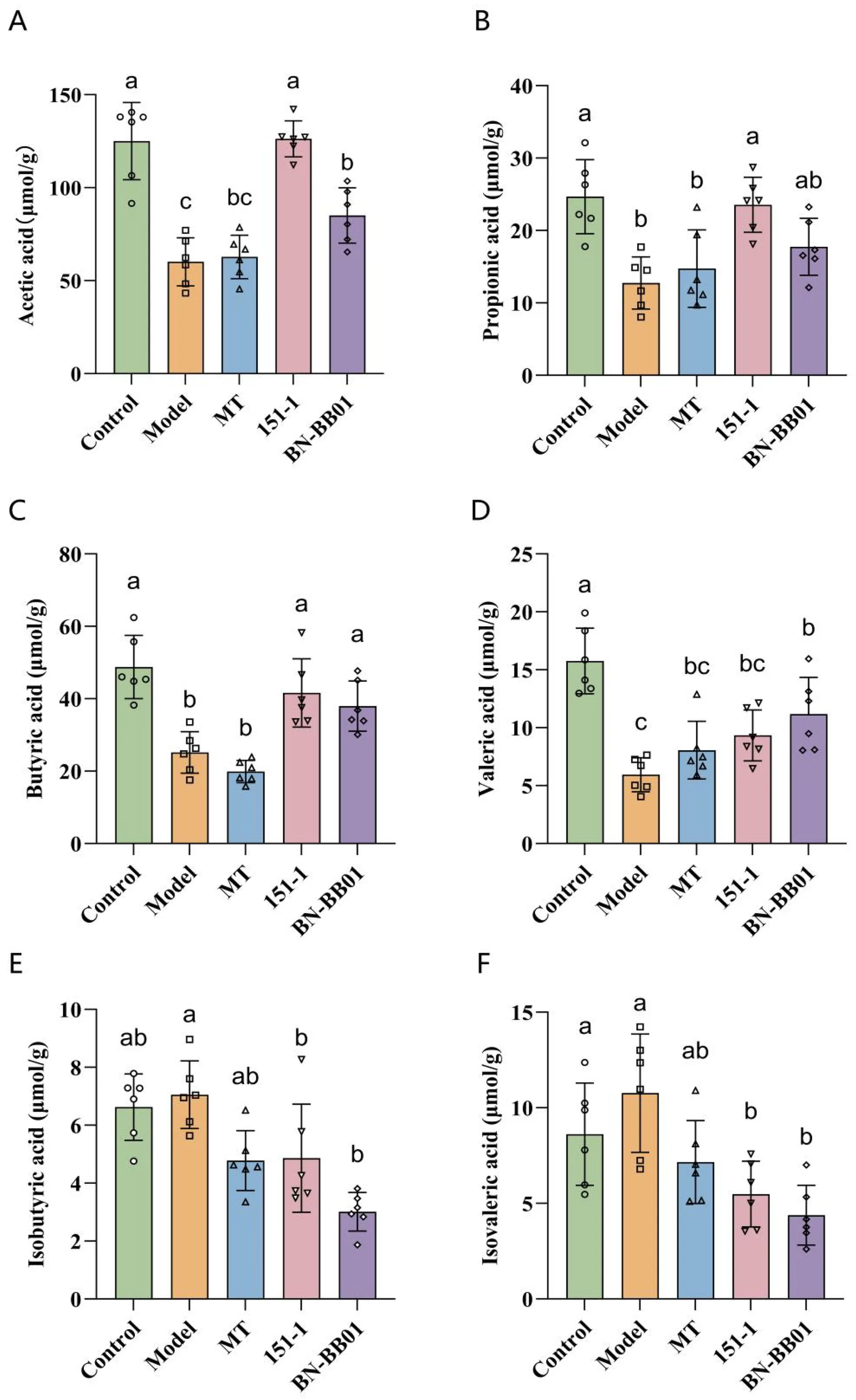
Determination of short-chain fatty acid contents in mouse feces: (**A**) acetic acid content, (**B**) propionic acid content, (**C**) butyric acid content, (**D**) valeric acid content, (**E**) isobutyric acid content, (**F**) isovaleric acid content. Different lowercase letters indicate significant differences among groups (*p* < 0.05).

Short-chain fatty acids (SCFAs) are key microbial metabolites that regulate intestinal motility and maintain gut health, playing critical roles in sustaining the intestinal barrier, immune homeostasis, gastrointestinal motility, and the gut–brain axis [21]. To evaluate the impact of the sleep deprivation-induced constipation model and probiotic intervention on gut microbiota metabolic function, the levels of short-chain fatty acids in mouse feces were determined in this experiment. As shown in Figure 5A-F, the contents of acetic acid, propionic acid, butyric acid, and valeric acid in the feces of the Model group were significantly lower than those in the normal control group, with the greatest change observed in acetic acid level, a decrease of 51.9% (*p* < 0.05). In contrast, the contents of isobutyric acid and isovaleric acid were higher than those in the control group. Overall, the total SCFA level was decreased, indicating that constipation in sleep-deprived mice inhibits the production of straight-chain SCFAs and promotes an increase in branched-chain SCFAs. After intervention with *B. longum* 151-1 and BN-BB01, the total SCFA level was higher than that in the Model group, with significantly increased contents of acetic acid, propionic acid, butyric acid, and valeric acid (*p* < 0.05). Compared with the Model group, acetic acid content increased by 66.14 μmol/g and 24.91 μmol/g, respectively; propionic acid content increased by 84.7% and 39.2%; butyric acid level increased by 65.4% and 50.9%; while the levels of isobutyric acid and isovaleric acid were significantly reduced (*p* < 0.05). In the positive drug MT group, propionic acid and butyric acid contents showed no significant change compared with the model group, and the effects on acetic acid and valeric acid levels were inferior to those of strains 151-1 and BN-BB01. The above results indicate that intervention with *B. longum* 151-1 and BN-BB01 in sleep-deprived constipated mice promotes the proliferation of beneficial bacteria, inhibits protein-putrefying bacteria, restores the capacity to produce straight-chain SCFAs, enhances intestinal epithelial energy supply, mucus secretion, and peristalsis promotion, thereby alleviating constipation; at the same time, it inhibits the proliferation of branched-chain SCFAs and reduces intestinal damage and irritation caused by putrefactive acids.

### 3.6. Regulation of Colonic Receptor Protein GPR41/GPR109A Signaling in Mice

**Figure 6:**
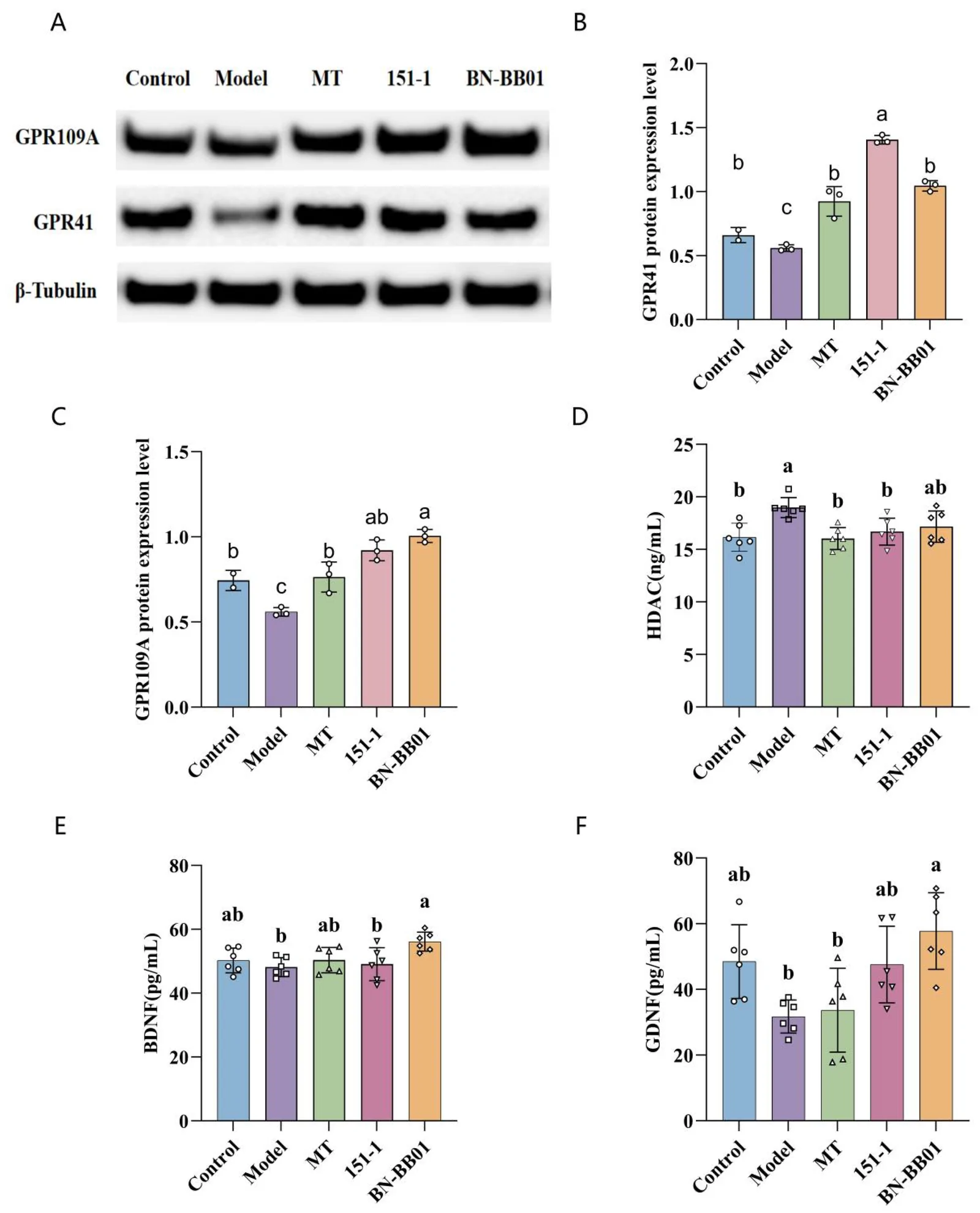
Expression of receptor proteins and neurotrophic factors in mouse colonic tissues: (**A**) Western blot bands of receptor proteins, (**B**) GPR41 protein expression level, (**C**) GPR109A protein expression level, (**D**) HDAC expression level, (**E**) BDNF expression level, (**F**) GDNF expression level. Different lowercase letters indicate significant differences among groups (*p* < 0.05).

Based on changes in fecal short-chain fatty acid (SCFA) levels, and to further explore the mechanism by which *B. longum* 151-1 and BN-BB01 alleviate constipation in sleep-deprived mice, this study focused on SCFA-mediated signaling pathways. The protein expression levels of GPR41 and GPR109A in colon tissue were quantitatively detected by Western blot (Figure 6A) to verify, at the receptor level, whether probiotic intervention could improve intestinal function through regulation of this pathway. The protein expression levels of both GPR109A and GPR41 in the colon tissue of the model group were significantly lower than those in the control group, indicating that short-chain fatty acid receptor signaling was markedly suppressed in sleep-deprived mice, which positively correlated with the reduced SCFA levels shown above. Quantitative analysis of band (using ImageJ software, Figure 6B,C) revealed that the expression levels of GPR41 and GPR109A receptors in the model group decreased by approximately 15.2% and 24.9%, respectively, compared with the control group. After intervention with the positive drug MT, *B. longum* 151-1, and BN-BB01, the expression levels of both receptor proteins increased to varying degrees. The 151-1 strain showed the most pronounced effect on the GPR41 receptor, while the BN-BB01 strain achieved a better recovery of GPR109A. This study indicates that *B. longum* 151-1 and BN-BB01 may restore SCFA levels and activate their receptor signaling, thereby enhancing gut–brain axis communication and promoting the recovery of intestinal motility.

### 3.7. Effects on Colonic Neurotrophic Factors and Epigenetic Regulators in Mice

HDAC, an important epigenetic regulator, is associated with impaired neuronal function due to its elevated expression, which inhibits the transcription of nerve-related genes. BDNF and GDNF are key neurotrophic factors that maintain enteric nervous system development and neuronal survival [22,23]. As shown in Figure 6D, HDAC levels in the colon tissue of the model group were elevated compared with the CK group, with an increase of approximately 17.5% (*p* < 0.05), while BDNF and GDNF levels showed decreasing trends, with reductions of approximately 4.2% and 34.6%, respectively (Figure 6E,F), suggesting that sleep deprivation may affect enteric nervous system and intestinal function by enhancing histone deacetylase activity and inhibiting neurotrophic factor expression. After intervention with strains 151-1 and BN-BB01, HDAC levels decreased compared with the model group, while GDNF and BDNF levels recovered to varying degrees, indicating that probiotic intervention may promote neurotrophic factor expression by modulating epigenetic regulators, thereby ameliorating sleep deprivation-induced enteric neural dysfunction. In contrast, the positive drug MT group showed a weaker effect, whereas the BN-BB01 group exhibited relatively more pronounced improvement. In summary, sleep deprivation may disrupt gut–brain axis homeostasis by affecting HDAC, BDNF, GDNF, and other molecules involved in enteric neural regulation. Intervention with *B. longum* 151-1 and BN-BB01 can alleviate constipation symptoms by modulating neural regulatory pathways and intestinal function.

### 3.8. Effects on the Gut Microbiota Composition in Mice

**Figure 7:**
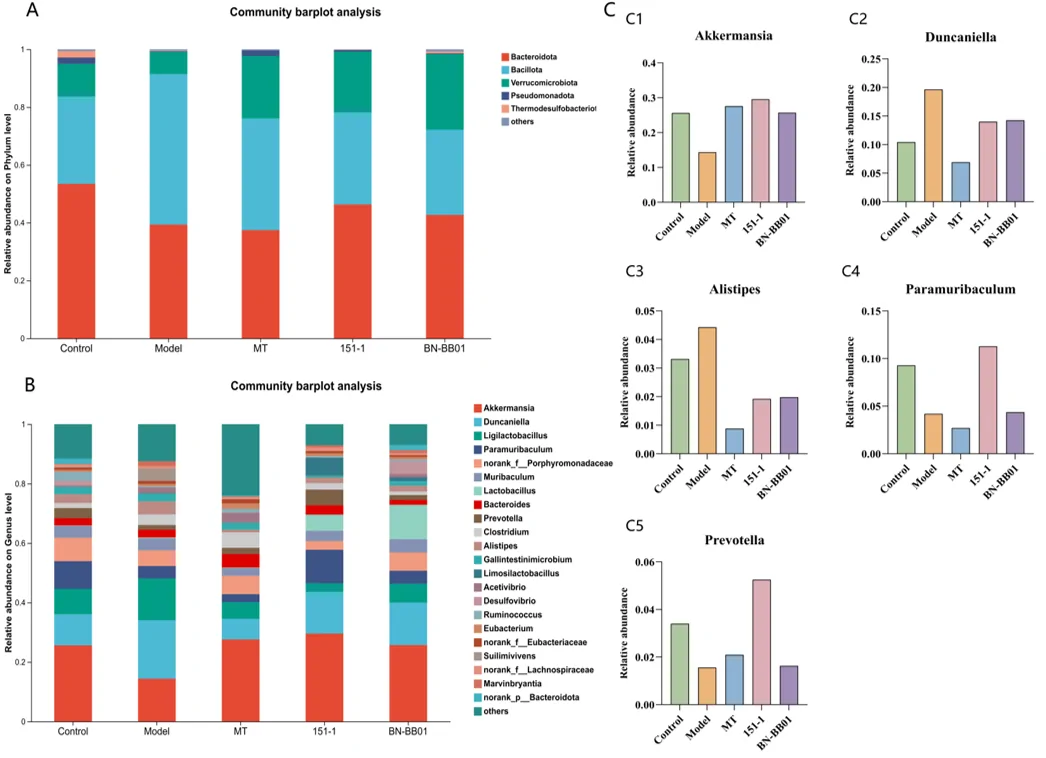
Species composition of fecal microbiota in mice: (**A**) species composition at the phylum level, (**B**) species composition at the genus level, (**C**) comparison of differential abundance at the genus level.

As shown in the phylum-level structural analysis of the five groups of mouse fecal microbiota in Figure 7A, the core microbiota were dominated by Bacteroidota and Firmicutes. The microbial composition of the normal control group was stable and consistent with the characteristic intestinal microbiota of healthy mice. The sleep deprivation model group exhibited significant gut microbiota dysbiosis, characterized by a decreased relative abundance of Bacteroidota, a marked increase in Firmicutes which became the dominant phylum, and a reduced abundance of Verrucomicrobiota. This pattern is consistent with previously reported sleep deprivation-induced gut microbiota dysbiosis, which is often accompanied by inflammation, intestinal barrier damage, and gut–brain axis dysfunction [24]. After intervention with *B. longum* 151-1 and BN-BB01, the microbial structure recovered toward that of the control group, with a significant increase in the abundance of Verrucomicrobiota, which promotes intestinal homeostasis repair. The melatonin group also showed similar improvement, although the effect was slightly weaker than that of the *Bifidobacterium* groups. Probiotic intervention regulated the proportion of dominant phyla and alleviated the imbalance between Bacteroidota and Firmicutes, which is an important indicator of significant microbiota improvement and exerts positive effects on the host.

Figure 7B shows the genus-level gut microbiota structure among the different groups. Compared with the CK group, the model group showed decreased relative abundances of genera such as *Bacteroides*, *Akkermansia*, *Paramuribaculum*, and *norank_f__Porphyromonadaceae*, and increased relative abundances of *Duncaniella* and *Alistipes*. Compared with the Model group, intervention with *B. longum* 151-1 and BN-BB01 increased the relative abundances of *Akkermansia*, *Lactobacillus*, and *Limosilactobacillus*, and decreased the relative abundances of *Duncaniella* and *Muribaculum*. After intervention with the positive drug MT, increased abundances of *Akkermansia* and *Bacteroides* and a decreased proportion of harmful bacteria such as *Alistipes* were observed. In summary, *B. longum* 151-1 and BN-BB01 ameliorated sleep deprivation-induced gut microbiota dysbiosis in constipated mice, increased the abundance of the adhesion-related beneficial bacterium *Akkermansia*, and decreased the abundance of the harmful intestinal bacterium *Alistipes*.

Differences in the relative abundances of gut microbiota at the genus level are shown in Figure 7C. There were certain compositional differences among the five treatment groups. Compared with the Control group, the Model group exhibited marked changes in the relative abundances of several genera, indicating that sleep deprivation affects the gut microbial composition of mice. The relative abundances of *Akkermansia* and *Prevotella* in the M group were lower than those in the CK group, while a trend toward recovery was observed in the *B. longum* 151-1, BN-BB01, and positive drug MT groups. For the genus *Alistipes*, the relative abundance was higher in the M group, whereas the probiotic intervention groups and the positive drug group showed decreases compared with the model group. For genera such as *Duncaniella* and *Paramuribaculum*, varying degrees of difference were observed among the groups, with fluctuations in the model group compared with the control group; after probiotic treatment, the overall trend moved toward the control group level. These findings indicate that strain intervention can ameliorate sleep deprivation-induced alterations in gut microbiota composition. By restoring SCFA-producing bacteria, the intervention activates the GPR41 and GPR109A receptor pathways and inhibits HDAC activity, thereby upregulating the levels of neurotrophic factors and neurotransmitters, and ultimately promoting the recovery of intestinal motility.

## 4. Discussion

Experimental studies of constipation have mostly used drug-induced slow-transit models [12,13], which are poorly suited to constipation that follows sleep loss and stress. Sleep deprivation can damage the intestinal barrier, disturb the microbiota, and promote neuroinflammation, yet previous work has seldom taken constipation as the primary endpoint and has lacked a systematic evaluation of *Bifidobacterium longum* acting through the gut–brain axis in this phenotype.

In the present study, sleep deprivation disrupted colonic mucosal architecture, reduced goblet cell numbers, and lowered intestinal transit, consistent with previously reported stress-induced barrier injury [25]. Barrier integrity depends on epithelial tight junctions and the mucus layer, and chronic stress can suppress peristalsis through activation of the HPA axis and the sympathetic nervous system [3,17,25]. Pang et al. likewise observed barrier disruption in sleep-deprived mice and showed that LGG restores the barrier and limits neuroinflammation via EGFR [8]. Gao et al. reported that melatonin attenuates corticosterone-related mucosal injury through the microbiota [5]. Our study focused on the constipation phenotype and motility, with melatonin as a positive control. Melatonin improved some transit indices but was weaker than the two *B. longum* strains for mucosal repair and several motility-related endocrine and metabolic endpoints, indicating that improving sleep-related chemistry is not equivalent to correcting sleep-related constipation. Lee et al. found that *B. longum* relieved stress-associated constipation in zebrafish by lowering cortisol and inflammation [10]; the direction of the stress-hormone change agrees with our data, but a zebrafish model cannot resolve colonic receptors or neurotrophic pathways in mammals. After *B. longum* intervention, intestinal structure improved markedly, suggesting that reinforcement of the mucosal barrier may provide structural support for the recovery of motility, in line with the view of Bischoff et al. that permeability is a therapeutic target [25]. Concurrent improvement at the study endpoint, however, does not prove that barrier repair precedes the recovery of motility.

Fecal acetate, propionate, and butyrate have been reported to be significantly lower in patients with insomnia than in controls, suggesting a link between sleep disturbance and microbial metabolic abnormalities [26]. This is consistent with the decrease in straight-chain SCFAs in our sleep-deprived mice and with reduced saccharolytic fermentation and increased protein fermentation [21,27]. Magzal et al. found that, in older adults with insomnia symptoms, sleep duration was associated with higher fecal SCFA concentrations [28]; diet, age, medication, and sleep efficiency may all modify the relationship between SCFAs and sleep. GPR41 and GPR109A are SCFA receptors: the former senses propionate and butyrate and participates in 5-HT release and sympathetic activity, whereas the latter is activated by butyrate and exerts anti-inflammatory effects [29,30]. The two strains were selected for different in vitro adhesion yet showed similar in vivo benefit, indicating that adhesion is not the sole determinant and that the SCFA–receptor pathway better explains their shared efficacy. At the same time, multi-platform water-environment sleep deprivation in mice is not equivalent to human insomnia, and SCFAs are only one link in the mechanistic chain.

Downstream of the receptors, epigenetic and neurotrophic changes transmit metabolic signals to the enteric nervous system. Gut metabolites during sleep deprivation can affect neurotrophic factor expression and have been linked to neuropsychiatric disorders and gastrointestinal dysmotility [22]. Among SCFAs, butyrate may upregulate BDNF and modulate enteric neuronal activity through HDAC inhibition [23]. Feedback between 5-HT and neurotrophic pathways can promote the growth and maturation of enteric nerve fibers and thereby alleviate functional intestinal disorders [31]. *Lacticaseibacillus paracasei* NCU-04 has been reported to improve constipation and depression-like behavior in mice and to restore microbial diversity by increasing colonic 5-HT synthesis and Htr4 expression [32]. We therefore treat the decrease in HDAC, the recovery of BDNF/GDNF, and the restoration of SCFAs and their receptors as interconnected evidence along the gut–brain axis.

Previous studies indicate that *Akkermansia muciniphila* is an important commensal that maintains barrier function, degrades polysaccharides, and contributes to acetate and propionate production [33]; *Bacteroides* and *Prevotella* are key polysaccharide-degrading, SCFA-producing taxa [27]; and expansion of *Alistipes* suppresses SCFA production [34]. The phylum-level shift after sleep deprivation (decreased Bacteroidota and increased Firmicutes) is consistent with reports on sleep and the microbiota and with sleep-deprivation studies in rats [6,24]. After intervention with *B. longum* 151-1 and BN-BB01, *Akkermansia* increased and *Alistipes* decreased, coinciding with recovery of straight-chain SCFAs, receptors, and neurotrophic markers, suggesting that remodeling of SCFA-producing and mucus-associated commensals may be an upstream event.

In summary, sleep deprivation may impair the mucus barrier and suppress motility through the HPA axis and microbial dysbiosis; SCFA-producing bacteria decline and straight-chain SCFAs fall; GPR41/GPR109A signaling weakens, HDAC is relatively increased, BDNF/GDNF are insufficient, and enteric excitatory/inhibitory balance is disturbed. The two *B. longum* strains may restore gut–brain axis function by remodeling the microbiota, recovering SCFAs and their receptors, inhibiting HDAC, and upregulating neurotrophic factors. This study has limitations. Constipation was induced only by multi-platform water-environment sleep deprivation and cannot be generalized to pharmacological, dietary, or idiopathic constipation. Only male mice were used, to avoid estrous-cycle effects on motility, stress hormones, and the microbiota; whether females respond similarly has not been tested. The proposed mechanism is supported by associations rather than causal evidence. Animal findings cannot be directly extrapolated to the clinic, but they provide candidate strains for adjunctive intervention in sleep-related gastrointestinal dysfunction; efficacy, safety, and optimal dosing still require clinical trials.

## 5. Conclusions

In this study, using a mouse model of emotional constipation induced by sleep deprivation, we systematically evaluated the intervention effects of *B. longum* 151-1 and BN-BB01 on constipation and explored the potential mechanisms by which they regulate constipation through the gut–brain axis. After intervention with the strains, the apparent constipation indicators in sleep-deprived mice were alleviated, with significantly upregulated levels of GAS, MTL, and SP, downregulated levels of VIP and CORT, and enhanced barrier repair function. SCFA levels (acetic acid, propionic acid, butyric acid) were increased, and the expression of the receptor proteins GPR41/109A was upregulated, which in turn inhibited HDAC activity, increased downstream BDNF and GDNF expression, and restored neurotransmitter homeostasis. The abundance of the beneficial bacterium *Akkermansia* in the gut microbiota was significantly increased, while the abundance of the harmful bacterium *Alistipes* was decreased, driving the activation of gut–brain axis signaling pathways from the upstream direction. This study provides new candidate strains and a mechanistic basis for the use of *B. longum* to improve sleep deprivation-related constipation.

## Data Availability

The raw experimental data supporting the conclusions of this article are available from the corresponding author upon reasonable request. The complete genome sequences of the bacterial strains used in this study have been deposited in GenBank: *Bifidobacterium longum* 151-1 under accession number JBZDSS000000000 (BioSample SAMN60678521), and *Bifidobacterium longum* BN-BB01 (recorded in the database as strain 60-2) under accession number JBZDSR000000000 (BioSample SAMN60678522). No human data or privacy restrictions apply.
